# The University of Kansas Cardiac Surgery Readmissions Committee: A Multidisciplinary Collaborative to Reduce Unplanned Readmissions

**DOI:** 10.1016/j.atssr.2025.07.012

**Published:** 2025-07-31

**Authors:** Regina Doonan, Kelsy Rice, Jessica Baldwin, Morgan Whisenhunt, Alicia Clarke, Kate Winkler, George Zorn, Todd C. Crawford

**Affiliations:** 1 Department of Thoracic and Cardiovascular Surgery, University of Kansas Hospital, Kansas City, Kansas; 2 Office of Quality and Safety, University of Kansas Hospital, Kansas City, Kansas

## Abstract

**Background:**

Unplanned readmissions negatively affect hospital reimbursement and mortality. The purpose of this study was to describe the inception of a multidisciplinary readmissions group designed to reduce unplanned cardiac surgery readmissions.

**Methods:**

Unplanned cardiac surgery readmissions within 30 days of discharge between 2021 and 2024 were analyzed by the University of Kansas Cardiac Surgery Readmissions Committee, consisting of inpatient and outpatient advanced practice providers, data managers, emergency department personnel, and cardiac surgeons. In 2023, a goal-oriented working group was established to reduce readmissions. Cardiac surgery readmissions are reviewed daily, and the readmissions committee meets quarterly to discuss their findings and opportunities for improvement.

**Results:**

The working group’s detailed investigation determined that arrhythmias, pleural effusions, and deep vein thrombosis or pulmonary embolism were the most common causes of readmission. The group then instituted postoperative amiodarone prophylaxis in their cardiac surgery patients, pursued aggressive postoperative drainage of the pleural spaces, and started prophylactic subcutaneous heparin on postoperative day 3 to reduce thromboembolic events. The group created a discharge checklist addressing rhythm issues, fluid balance, wound care, and clinic and on-call contact information. From 2021 to 2024, cardiac surgery readmissions declined from 10.9% to 9.6%, and similarly isolated readmissions after coronary artery bypass grafting declined from 10.2% to 6.1%, despite an increase in total operative volume and an increased case-mix index.

**Conclusions:**

The creation of an integrated, multidisciplinary Cardiac Surgery Readmissions Committee Working Group allowed the group to design targeted interventions for the most common reasons for readmission, which in turn led to a reduction in readmission rates.

## Visual Abstract

**Figure:**
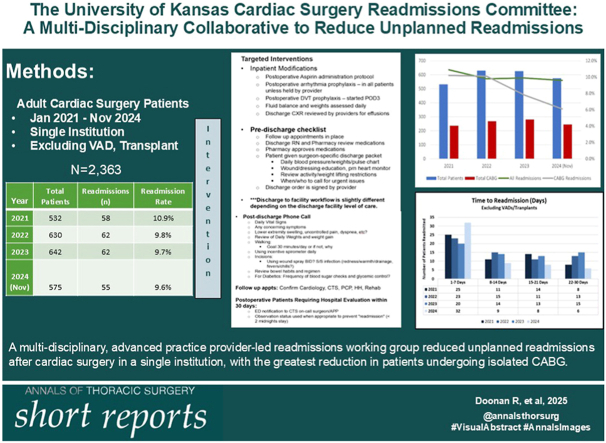

**In Short**

▪ A multidisciplinary readmissions group can reduce unplanned cardiac surgery readmissions.
▪ A predischarge checklist and a postdischarge follow-up telephone call can identify patients at risk for readmission.
▪ Integration between inpatient and outpatient provider teams fosters improved patient care and a better patient experience.

Cardiac surgery readmissions have emerged as an intriguing quality metric. In addition to the financial burden readmissions place on a health system, they also are an independent risk factor for all-cause mortality.^1^ In 2009, a study published in the *New England Journal of Medicine* shed light on the financial impact of readmissions after publishing that among Medicare beneficiaries in 2004, 20% of patients were readmitted within 30 days, at a cost of $17.4 billion.^2^ Introduced in 2012 as part of the Affordable Care Act, the Centers for Medicare & Medicaid Services Readmissions Reduction Program began penalizing institutions with excessive 30-day readmission rate for the diagnoses of heart failure, myocardial infarction, and pneumonia. Payers, including the Centers for Medicare & Medicaid Services, now use the excess readmit ratio to assess hospital performance, and coronary artery bypass grafting (CABG) is one of the conditions used in the excess readmit ratio calculation.^3^ Average cost per readmission across all payers according to the Agency for Healthcare Research and Quality was $15,200 in 2018, and among cardiac surgical reasons for readmission, including endocarditis, valve disorders, and aortic aneurysms, the average cost was substantially greater.^4^

Although most studies pertaining to cardiac surgery readmissions are institution specific, these studies suggest that readmission rates approach 10% to 20%.^5^, ^6^, ^7^ Efforts to reduce unplanned readmissions after cardiac surgery have taken shape across the country, including our own institution (University of Kansas Hospital, Kansas City, KS). Here we describe the inception and evolution of a Cardiac Surgery Readmissions Committee Working Group, designed to identify common reasons for readmission, institute safe and reasonable strategies to reduce unplanned readmissions, and identify potential obstacles to successful implementation of these strategies.

## Patients and Methods

### Readmission

Cardiac surgery readmission was identified as any unplanned readmission to any hospital within 30 days after discharge among patients who underwent cardiac surgery at the University of Kansas Hospital. Readmission was qualified by an inpatient admission order, which was specific to each hospital. Patients who were admitted for observation and did not meet criteria for inpatient status were not captured as readmissions. Notably, at our institution, patients admitted under observation who spend more than 2 nights in the hospital are upgraded to inpatient status.

### Patients

The Cardiac Surgery Readmissions Committee reviews all patients readmitted after undergoing open cardiac surgery operations, including the following: CABG; aortic, mitral, pulmonic, or tricuspid valve repair or replacement; arrhythmia surgery; aortic surgery; pulmonary or caval thrombectomy or embolectomy; or septal myectomy. Because this is a quality initiative with aggregate, deidentified data, a waiver from informed consent was granted by the Institutional Review Board.

### Cardiac Surgery Readmissions Committee

Formed in 2016, the University of Kansas Hospital Cardiac Surgery Readmissions Committee comprises inpatient and outpatient advanced practice providers, data managers (including our quality outcomes coordinator), faculty cardiac surgeons, cardiac surgery fellows, clinic nurses, and discharge nurse coordinators. The readmissions committee meets quarterly to review all postoperative readmissions and to discuss readmission trends, common reasons for readmission, and potential targets for intervention. We review our readmissions data quarterly at our departmental quality assurance meeting to assess progress. In response, several important initiatives have taken shape on the inpatient side: (1) postoperative initiation of amiodarone prophylaxis for all cardiac surgery patients unless contraindicated, (2) starting thromboembolic chemoprophylaxis with subcutaneous heparin on postoperative day 3, and (3) daily assessment of fluid balance on the basis of fluid intake and output and weight tracking. Our discharge nurse coordinator also completes a predischarge checklist with every patient (Supplemental Table 1). On the outpatient side, clinic nurses make early telephone calls between postdischarge days 3 and 5 to review vital signs, weight changes, and wound healing (Supplemental Table 2). Finally, we have prioritized closed-loop communication among front-line emergency medicine and cardiology providers when patients present back to the hospital within 30 days of discharge.

### Data Analysis

All readmission data are collected and compiled by our unblinded quality outcomes coordinator. Readmission rates are calculated for the entire open cardiac surgery cohort, as well as for specific index operations such as isolated CABG and isolated aortic valve replacement. The annual case-mix index (CMI), calculated from the average Medicare Severity-Diagnosis Related Group weight of hospital discharges, is longitudinally compared with our annual readmission rates. Descriptive statistics were used, and categorical variables were listed as n (percentage).

## Results

**Figure 1:**
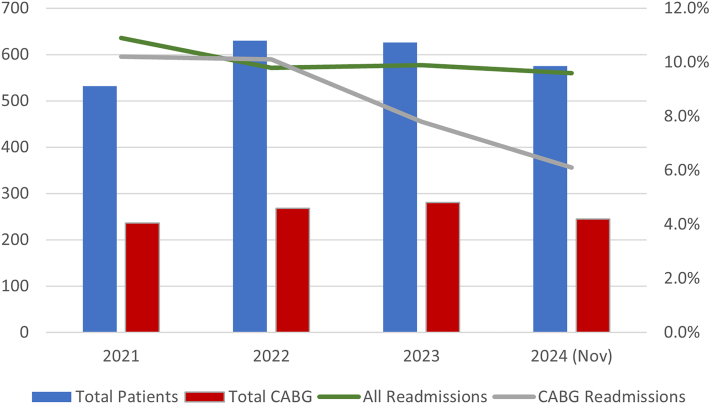
Comparison of cardiac surgery readmission rates from 2021 to 2024. (CABG, coronary artery bypass grafting.)

Readmission rates over the years 2021 to 2024 at our institution were 10.9% (58 of 532 patients) in 2021, 9.8% (62 of 630) in 2022, 9.9% (62 of 626) in 2023, and 9.6% (55 of 575) through November 2024 (Table 1). Readmission rates in patients who underwent isolated CABG steadily decreased from 10.2% (24 of 236) in 2021 to 7.8% (22 of 81) in 2023 to 6.1% (15 of 245) through November 2024 (Figure 1). The highest readmission rates by operation were observed in the combined aortic and mitral valve replacement group followed by mitral repair or replacement with CABG. Among patients with CMI data available, the CMI remained high over the 3 years from 7.296 in 2022 to 7.085 in 2024.

**Table 1:** Longitudinal Assessment of Cardiac Surgery Readmission Rates, 2021 to 2024

Year	Total Patients, n	Readmissions, n	Readmission Rate, %
2021	532	58	10.9
2022	630	62	9.8
2023	642	62	9.7
2024 (Nov)	575	55	9.6

**Figure 2:**
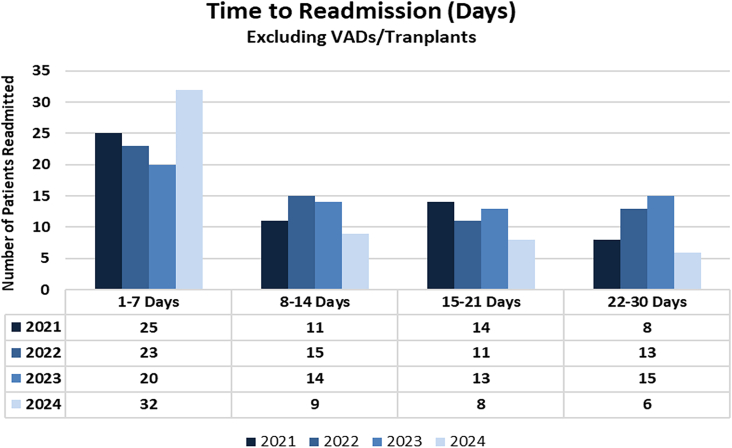
Timing of 30-day readmissions from 2021 to 2024. (VADs, ventricular assist devices.)

A majority of readmissions happened within 14 days of discharge with the highest rate of readmission occurring in the first 7 days after discharge (Figure 2). This trend has persisted over the previous 3 years and was even further amplified in 2024, despite increased awareness and attention to readmissions.

The most common reasons for postoperative readmission over the last 3 years include arrhythmias, pleural effusions, and gastrointestinal issues followed by congestive heart failure and wounds (Table 2).

**Table 2:** Longitudinal Assessment of Reasons for Readmission, 2021 to 2024

Readmission Reasons	Year			
	2021	2022	2023	2024
Aortic complication	0.0	1.6	3.2	1.9
Arryhthmia/heart block	22.4	12.9	21.0	16.7
Blood pressure	3.4	0.0	0.0	3.7
CHF	6.9	9.7	3.2	5.6
GI issues	12.1	3.2	4.8	16.7
DSI/SSI	1.7	4.8	1.6	1.9
MI/chest pain	0.0	1.6	3.2	3.7
Pericardial/pleural effusion	13.8	19.4	14.5	22.2
PE/DVT	5.2	1.6	11.3	1.9
Pneumonia/respiratory	5.2	3.2	4.8	3.7
Sepsis	6.9	0.0	1.6	0.0
TIA/stroke	5.2	1.6	4.8	0.0
Wound/vascular	8.6	11.3	3.2	7.4
Other, related	1.7	6.5	9.7	7.4
Other, nonrelated	6.9	22.6	12.9	7.4

CHF, congestive heart failure; DSI, deep space infection; DVT, deep vein thrombosis; GI, gastrointestinal; MI, myocardial infarction; PE, pulmonary embolism; SSI, surgical site infection; TIA, transient ischemic attack.

After the Readmissions Committee Working Group instituted targeted interventions in 2023 including postoperative amiodarone prophylaxis, early initiation of deep vein thrombosis prophylaxis, and aggressive drainage of the pleural space and careful attention to postoperative fluid balance, we recognized immediate improvements in readmission rates. This is best captured by our 40% reduction in readmission rates in our isolated CABG population and a modest but sustained reduction in overall readmission rates for our cardiac surgery population over the last four years.

## Comment

Our results demonstrate that an advanced practice provider–led multidisciplinary working group led to a reduction in cardiac surgery readmission rates over several years despite a sustained high level of patient complexity. A prospective, proactive approach to all cardiac surgery readmissions enabled our group to drill down on common reasons for readmission and to enact timely interventions to prevent these reasons from occurring.

Postoperative cardiac readmission rates at our institution ranged between 9% and 10% over the last 4 years, and we recognized considerable improvement in our isolated CABG readmissions over this time period. In comparison with recent publications, our working group’s careful attention to common causes of readmission led to our slightly lower readmission rates compared with those published in single-institution and multiinstitutional studies.^5^^,^^8^ One previous single-institution study from 2012 determined that the median time to readmission after discharge from CABG was 6 days.^9^ Earlier readmission was also identified in a recent 10-center cohort study where a majority (∼50%) of cardiac surgery readmissions occurred within the first few weeks after discharge.^7^ Certainly, we have identified the first 7 days as the most vulnerable period for our postoperative patients, with the highest percentage of readmissions occurring within the first week. Because our catchment area is vast, we have encouraged patients to stay in our greater metropolitan area for the first week after discharge to potentially reduce local emergency department visits and unnecessary readmissions.

Fluid accumulation, either in the pleural or pericardial space, and arrhythmias were the most common categorized reasons for readmission, similar to what has previously been published.^8^ However, deep vein thrombosis or pulmonary emboli and gastrointestinal complications, including bleeding, have recently emerged as more common reasons for readmission at our institution. This is a temporal change from wound infections and heart failure, which were formerly the more common factors affecting readmission. One previous study looking across the state of New York also identified postoperative infection and heart failure as the most common reasons for readmission.^7^ We recognize that timing of postoperative chemoprophylaxis initation for venous thromboembolism is controversial, and our surgeons achieved consensus on initiating chemoprophylaxis on postoperative day 3 for all patients.

A comprehensive discharge checklist and close postoperative follow-up by our outpatient nurses and advanced practice providers with attention to vital sign trends, weight gain, and other symptoms of fluid retention likely had a positive impact on our readmission rates over the study period. This protocol is a crucial component of our Readmissions Committee Working Group. In fact, other institutions have also demonstrated that establishing an advanced practice provider–led home transitional care program with home visits had the greatest impact on reducing hospital readmissions after CABG.^10^

Because our study reflects the findings of only our institution, it has several limitations. One potential limitation is that although our working group attempts to capture all known readmissions, we are reliant on feedback from admitting facilities or the patient to inform us of outside readmissions. Thus, our readmission rate could be underestimated if outside hospital readmissions are uncaptured. This issue is important because a recent study identified that readmission to nonindex institutions is associated with significantly higher mortality.^8^ Additionally, observer bias may have contributed to our reduction in readmissions. We are now more aware of the burden of readmissions and have been more diligent in managing some common postoperative complications in the outpatient setting or admitting patients for observation, which is associated with a reduced duration of readmission, thus improving the patient’s experience and reducing hospital costs.

Taken together, our findings suggest that a multidisciplinary, proactive approach to readmissions may lead to successful reduction in cardiac surgery readmissions over time. Because readmissions negatively affect patients’ outcomes and are increasingly scrutinized by regulatory bodies and payers, we must not lose sight of the big picture. Moving forward, efforts to reduce unplanned readmissions must prioritize high-quality care for the patient and improve the patient’s experience, first and foremost, rather than simply seek to improve the profitability of medical institutions. This task will not come easy, given the increasing complexity of patients presenting for cardiac surgery. Integrating inpatient and outpatient providers within a readmissions committee, meeting frequently to assess common readmission reasons, and identifying opportunities for improvement are easy, effective, and proactive ways to embrace this ongoing challenge for health systems and cardiac surgery programs.
