# Supplementary material for: The University of Kansas Cardiac Surgery Readmissions Committee: A Multidisciplinary Collaborative to Reduce Unplanned Readmissions
Source: Ann Thorac Surg Short Rep. 2025 Jul 31;4(1):340–5. doi: 10.1016/j.atssr.2025.07.012 (PMC13100770; doi:10.1016/j.atssr.2025.07.012)
Supplement: Supplemental Table 1 [file mmc1.docx]

**Supplemental Table 1**

| **Targeted Interventions** |
| --- |
| - **Inpatient Modifications** - **Postoperative Aspirin administration protocol** - **Postoperative arrhythmia prophylaxis – in all patients unless held by provider** - **Postoperative DVT prophylaxis – started POD3** - **Fluid balance and weights assessed daily** - **Discharge CXR reviewed by providers for effusions** |
| - **Pre-Discharge Checklist** - **Follow up appointments in place** - **Discharge RN and Pharmacy review medications** - **Pharmacy approves medications** - **Patient given surgeon-specific discharge packet**   **Daily blood pressure/weights/pulse chart**  **Wound/dressing education, prn heart monitor**  **Review activity/weight lifting restrictions**  **When/who to call for urgent issues**   - **Discharge order is signed by provider** - *****Discharge to facility workflow is slightly different depending on level of care** |
