# Supplementary material for: The University of Kansas Cardiac Surgery Readmissions Committee: A Multidisciplinary Collaborative to Reduce Unplanned Readmissions
Source: Ann Thorac Surg Short Rep. 2025 Jul 31;4(1):340–5. doi: 10.1016/j.atssr.2025.07.012 (PMC13100770; doi:10.1016/j.atssr.2025.07.012)
Supplement: Supplemental Table 2 [file mmc2.docx]

**Supplemental Table 2**

| **Post-discharge Phone Call**   - **Daily Vital Signs** - **Any concerning symptoms** - **Lower extremity swelling, uncontrolled pain, dyspnea, etc** - **Review of Daily Weights and weight gain** - **Walking:**   **Goal 30 minutes/day or if not, why**   - **Using incentive spirometer daily** - **Incisions:**   **Using wound spray bid? Symptoms/signs of infection - redness, warmth, drainage, fevers or chills?)**   - **Review bowel habits and regimen** - **For Diabetics: Frequency of blood sugar checks and glycemic control?**   **Follow-up Appointments: Confirm Cardiology, CTS, Primary Care, Home Health, Rehab**  **Postoperative Patients Requiring Hospital Evaluation within 30 days:**   - **Emergency Department notification to CTS on-call surgeon or APP** - **Observation status used when appropriate (>2 midnights stay)** |
| --- |
